# The reasons why Pakistan might be at high risk of Crimean Congo haemorrhagic fever epidemic; a scoping review of the literature

**DOI:** 10.1186/s12985-017-0726-4

**Published:** 2017-03-23

**Authors:** Muhammad Atif, Anum Saqib, Raazeyah Ikram, Muhammad Rehan Sarwar, Shane Scahill

**Affiliations:** 1 The Islamia University of Bahawalpur, Bahawalpur, Punjab Pakistan; 2 Massey University, Auckland, New Zealand

**Keywords:** Crimean Congo Haemorrhagic Fever, Crimean Congo Haemorrhagic Fever Virus, CCHF, CCHFV, Pakistan, Eid-ul-Azha

## Abstract

Pakistan has faced a number of significant healthcare challenges over the past decade. In 2000, one of these events - a deadly epidemic of Crimean Congo Haemorrhagic Fever (CCHF) - struck Pakistan. The people of Pakistan are at a very high risk of acquiring CCHF, due to a number of factors which emerge from a scoping review of the literature. First, the underdeveloped healthcare system of the country is currently not prepared to cope with challenges of this nature. Healthcare professionals and medical institutes are not sufficiently equipped to properly diagnose, manage and prevent CCHF. Second, a large percentage of the general public is unaware of the spread and control of the vector. The agricultural sector of Pakistan is vast and thus many people are involved in animal husbandry and the handling of livestock which can lead to the transmission of the CCHF virus. Even in urban areas the risk of transmission is significantly higher around the time of Eid-ul-Azha, when Muslims slaughter animals. Finally, the political upheavals faced by the country have also increased Pakistan’s vulnerability because a large number of refugees from Afghanistan, a CCHF endemic country, have migrated to Pakistan as a result of the Afghan war. Most of the refugees and their animals settle in Baluchistan and Khyber Pakhtunkhwa provinces, which consequently have a higher prevalence of CCHF. This scoping review of the literature highlights the potential causes of high risk CCHF and draws conclusions and makes recommendations that policy-makers in Pakistan may wish to consider in-order to improve on the current situation.

## Background

Crimean Congo Haemorrhagic Fever (CCHF) is a potentially fatal tick-borne zoonosis characterized by haemorrhage, with a fatality rate of between 10 and 50% [1]. The causative organism of CCHF - Crimean Congo Haemorrhagic Fever Virus (CCHFV) - belongs to the family *Bunyaviridae*, and genus *Nairovirus* and is the world’s most prevalent tick-borne virus [2]. The tick which is the focal point for the transmission of CCHFV belongs to the genus *Hyalomma*. The environmental conditions in Pakistan are optimal for the replication of ticks, and this further increases the chances of spread of CCHFV infection [3].

Basic medical amenities are available in the healthcare system in urban areas of Pakistan, but unfortunately this is not consistent throughout the whole country [4]. As far as the rural areas are concerned, there is a dearth of Basic Healthcare Units (BHU). According to one estimate, 127,859 doctors and 12,804 healthcare facilities are present in Pakistan to serve a population of more than 170 million people [5]. In 2007, 85 physicians were available to serve every 100,000 patients, equating to 1,225 patients per doctor [6].

During the recent past, the public health sector of Pakistan has been challenged by a series of epidemics and this is ongoing. Once the outbreak of polio and dengue fever was successfully under control, CCHF emerged as a significant public health concern. This put undue pressure on the health system for sustained periods of time. Pakistan was recognized as a victim of CCHF as early as 1976 when the first case was reported in Rawalpindi General Hospital [7] and subsequently it became endemic in Pakistan by the year 2000 [8, 9].

The CCHF virus is transmitted to humans through tick bites or by direct contact with infected animal blood often via farmers, slaughterhouse workers, veterinarians and so forth. In addition, person-to-person transmission can then occur due to direct or indirect contact with the skin, mucous membranes, or body fluids of infected patients [10]. Tick bites serve as the major mode of transmission of disease; however, the blood, body fluids and tissues of susceptible animals might also pose a threat for humans [11]. This viral disease remains asymptomatic in animals [12] and poses a threat to people from across the population. For instance, healthcare professionals who deal with patients infected with CCHFV are at a high risk of infection due to inadequate biosafety policies and practices and a lack of proper management procedures as a result [3, 8, 9]. Agriculture is a main source of livelihood for a large number of people in Pakistan, particularly those residing in villages and towns and this involves extensive animal husbandry and livestock management. Since a large number of vectors responsible for entomological diseases and endemic pathogens are present in agricultural areas in Pakistan, so people are more prone to CCHF exposure. Linked to this dominance of the agriculture sector, livestock is a major capital consideration in rural Pakistan and animal husbandry is the major source of income for around three quarters of the Pakistani population. As such, people are always in contact with animals and their exposure to and risk of zoonotic diseases is greatly increased because of this. Moreover, the frequent shifting of nomads with their livestock [13, 14] plays a significant role in the recurring epidemics of CCHFV in Baluchistan and neighbouring Afghanistan. Internationally studies [2, 15, 16] have reported the epidemiological profile of CCHFV in domestic and wild animals but this data is not available for Pakistan. The National Institute of Health (NIH) of Pakistan is an autonomous body which works in collaboration with the WHO to monitor CCHFV cases on a daily basis and provides feedback to the relevant health department [17].

**Fig. 1:**
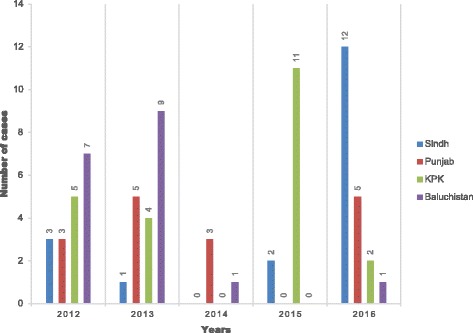
Death Toll of Crimean Congo Haemorrhagic Fever cases in Pakistan during 2012-2016 [13, 18, 19]

In 2016 a total of 20 patients died in Pakistan from CCHF [18], the majority being from the Sindh province. However, mortality data since 2012 suggests significant variation in where deaths occur by province by year with no apparent pattern (Fig. 1).

In line with a lack of mortality pattern there is no appropriate arrangement for monitoring the outbreak and spread of CCHF in Pakistan. The approach is largely reactive and relies almost exclusively upon the assessment of individual cases related to location, prevalence and lethality [3]. Therefore the objective of this scoping review is to identify the reasons for which Pakistan is at a high risk of CCHFV outbreak.

## Search strategy and review methodology

### Type of literature review

There are many different options for conducting literature reviews and this is a scoping review of the literature. Scoping reviews provide for theoretically informed discussions of the “big issues” in a topic area that has not been well studied in a particular context. To do this a broad range of literature in the CCHF field is reviewed and analysed for key issues. To achieve our objective, the search of the literature included those papers that discuss and highlight the pertinent issues. Scoping reviews often contain research questions that are broad and the inclusion/exclusion criteria is often developed post hoc. Quality of publication is initially not a priority as an understanding of the overall discourse is to be attained. Raw data extraction may or may not be required and in this study raw data is not analysed. The synthesis is more qualitative than quantitative and this is demonstrated by the development of key themes which identify parameters and significant gaps in the body of literature [19].

Therefore the scoping literature review fits nicely as a method for this study as it involves a process of “summarizing a range of evidence in order to convey the breadth and depth of a field” [20]. The aim is to map the key concepts underpinning a research area and the main sources and evidence available; in this case CCHF [21]. Importantly, scoping reviews can be undertaken as stand-alone projects in their own right, especially where an area like CCHF which is complex, or the literature has not been reviewed comprehensively before [22]. There are five phases to a scoping reviews and these steps were followed in this study [22]:

1. Identifying the research question
2. Identifying relevant studies
3. Study selection
4. Charting the data
5. Collating, summarising and reporting the results

### Search strategy

A comprehensive literature search (studies published between 1^st^ January 2000 and 31^st^ October 2016) was generated using Embase, EconLit, Google Scholar, Medline, PubMed, ProQuest, Scopus, Springer Link and Science Direct databases. Search terms included “Tick”, “Viral infections”, “Hemorrhagic fever”, “Nosocomial”, “Crimean Congo hemorrhagic fever”, “Crimean Congo hemorrhagic fever virus”, “CCHF”, “CCHFV” and “Eid-ul-Azha”. The keywords were used in diverse combinations with BOOLEAN and MeSH searches. Further publications were recognized by a manual search of references of related papers, and review articles were also cited where applicable. Initially, 154 studies were retrieved. Out of these, 52 were duplicates and therefore were excluded. Out of 102 remaining articles, 3 were excluded based on irrelevant titles and/or abstracts. Subsequently, full text of 99 articles were read and 80 studies were further removed as these failed to provide relevant details. Finally, 19 articles were selected based on the inclusion and exclusion criteria (Table 1) with all the authors agreeing to include these studies in the scoping review. Some important sources such as NIH website, WHO website and web page of local news papers were also searched to retrieve the relevant data.

**Table 1:** Inclusion and exclusion criteria

Sr. No	Inclusion criteria
1	Studies on CCHFV infection published from 1^st^ January 2000 to 31^st^ October 2016.
2	All original studies, reviews and abstracts available in scientific literature.
Sr. No	Exclusion criteria
1	Studies published in a language other than English were excluded.

In this scoping review, the literature was thematically analysed and presented as a series of broad themes including: ticks and the environment, the health care system, a rural community, livestock and Eid-ul-Azha and nomadic life-style.

### Data collection

A purpose designed data extraction form was used to identify the themes that emerged from the literature. Each record described the potential reasons for why Pakistan is at high risk of CCHFV infection. All entered data were rechecked and Microsoft Excel 2010 was used for the category analysis of the themes as follows.

### Thematic analysis

After the extraction of themes from categorising of the literature a review was undertaken to ensure that the emergent themes were sourced from the most complete collection of relevant literature possible. In this scoping review the aim is to understand the broad yet significant issues that emerge from the literature as to why Pakistan is more prone to CCHFV infection.

## Findings and discussion

From the scoping review emerged the following themes which represent the potential reasons why Pakistan is at high risk of CCHF epidemics: ticks and the environment, the health care system, a rural community, Livestock and Eid-ul-Azha and nomadic life-style. These themes are presented and discussed each in turn below:

### Ticks and the environment

**Fig. 2:**
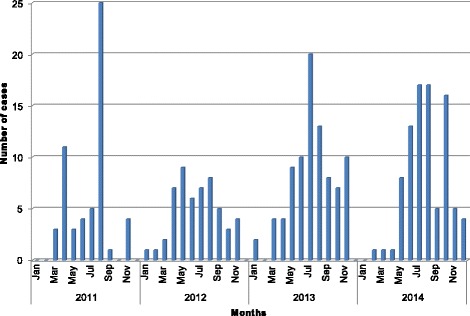
Biannual surge in CCHFV infection cases between March to May and July to September during 2011-2014 in Pakistan [29]

Ticks of the genus *Hyalomma* act both as vectors and reservoirs for CCHFV [23]. CCHF occurs most commonly in geographical areas such as Pakistan; which are rich in vegetation and possess warm weather [24, 25]. Pakistan provides an excellent habitat for ticks due to these optimal weather conditions including; mild winters and warm autumns, low rainfall, and changes in the cultivation of land and its use. Such environments are optimal for the activation of *Hyalomma* ticks [24]. As the temperature increases, particularly toward the end of autumn, the ticks begin to moult at a rapid rate enabling them to change into an adult. Due to this, the number of infections reported rises during March to May and then again between July and September (Fig. 2) [26]. Mild winters are favourable for ticks, thereby increasing their survival and the population rate. Moreover, the geographic pattern of CCHF outbreaks in Pakistan suggests a relationship between CCHF and altitude. A study from Turkey supports this by reporting that people living in rural areas and working in animal husbandries at altitudes greater than 600m in May, June and July, are at higher risk of contracting CCHF [27]. In summary, the environmental conditions within Pakistan means residents of this country are more prone to CCHFV infection [25].

### The healthcare system

Unfortunately, the Pakistani healthcare system is not prepared to cope with CCHFV epidemics and does not have the capacity [28] to deal with this significant public health issue for a number of reasons [29]. First, there is a lack of trained staff and the professional competency of healthcare professionals is lower than that required to deal with CCHF [4, 30]. Since staff is inadequately trained they lack the knowledge to be able to respond promptly in the case of disease outbreak [29]. As is often the case, instead of dealing with the infection at hand on an urgent basis, healthcare professionals refrain from treating CCHF infected patients for fear of contracting the disease themselves [29]. Second, there is a shortage of essential medicines and appropriate laboratory equipment which is essential for the diagnosis of CCHF [29]. Prompt diagnosis facilitates the timely management and reduces further transmission of CCHFV [31]. Third, the absence of quarantine areas or infection control policies is likely to worsen the situation in Pakistan [4]. Finally, there is a lack of proper contact tracing procedures for both patients and staff which is also expected to reduce the morbidity and mortality rates in the long term.

Those of high socio-economic standing working within urban health care facilities are not immune to the impact of this viral disease. CCHFV infection led to the death of a senior surgeon at the Bahawalpur Victoria Hospital (BVH) (located in Bahawalpur, Punjab province of Pakistan) on 30^th^ July 2016. He acquired the infection during a surgical procedure of a patient (who had previous contact with livestock) suffering from haemorrhagic peritonitis. The lack of precautionary measures at BVH, inadequate knowledge by healthcare professionals and absence of a proper diagnosis resulted in the death of the imminent surgeon. Nosocomial infections arise due to the inadequacies in provision of the most basic services in an otherwise stretched Pakistani healthcare system. In an ideal world doctors should be very well prepared and educated about the prevention, diagnosis and management of CCHF so that they can provide high quality care to the patients all the while protecting themselves [32]. Unfortunately this does not appear to be common practice in Pakistan and this is borne out in the literature.

### A rural community

In Pakistan the population is comprised of approximately one-third urban (36%) and two-thirds rural (64%). There are a number of factors which place both of these populations, especially the rural population, at high risk of acquiring CCHF. First, Pakistan is a developing country with a very low literacy rate. Specifically, most people live in rural areas who are in direct contact with livestock are illiterate. Lack of knowledge and awareness among the animal handlers contributes significantly to the rapid spread of CCHF [33]. Moreover, due to poverty, many of the animal handlers are unable to attain animal vaccines or maintain conditions of proper hygiene for their livestock. This puts them at a very high risk of infection themselves whilst also being the source of ongoing transmission of CCHF to other humans.

In addition to the rural folk who keep animals, urban and rural butchers are another high risk group for CCHF. There are countless meat shops in every city in Pakistan but very few maintain conditions including proper hygiene that would be expected to reduce CCHF transmission. These shops are open aired stalls where the butcher chops the meat, barehanded, in front of the customers, while the un-skinned, whole bodies of animals hang in front of the stalls. Without gloves, the butchers can easily get infected through minor cuts while chopping the meat of an infected animal.

The potential high risk groups of CCHF are recognized not only by their professional or work roles but also geographically. The prevalence of CCHF is highest in Baluchistan, Khyber Pakhtunkhwa (KPK; where KPK is one of the four provinces of Pakistan, located in the north-western region of the country. It was formerly known as the North-West Frontier Province (NWFP) which is commonly called *Sarhad*) and the Federally Administered Tribal Areas (FATA; where FATA is a semi-autonomous tribal region in north-western Pakistan, bordering Pakistan’s provinces of Khyber Pakhtunkhwa and Balochistan to the east and south, and Afghanistan’s provinces of Kunar, Nangarhar, Paktia, Khost and Paktika to the west and north) of Pakistan (Table 2). These regions share borders with CCHF endemic countries such as Iran and Afghanistan [4, 26, 34]. While a favourable environment and climate for the ticks is a reason for the geographic distribution of CCHF, the greatest contributor to the epidemic is likely to be the immigration of Afghan refugees from across the border [35]. CCHF has been reported to have a high prevalence in Afghanistan [36] and so the settling of Afghanis in Baluchistan and KPK continues to be a significant source of transmission of the virus. People living in Afghanistan also visit the border city of Quetta in Pakistan to attend healthcare facilities and CCHF is often detected in these patients as well.

**Table 2:** IgM confirmed Crimean Congo Haemorrhagic Fever cases found during the years 2003 to 2008 reported to National Institute of Health, Islamabad, Pakistan [4]

Areas according to the disease burden	Provinces	Number of IgM confirmed cases
High burden areas	Baluchistan	57
	Khyber Pakhtunkhwa	20
Low burden areas	Punjab	6
	Sindh^a^	2

^a^Low number of cases in the Sindh province may be either due to low prevalence of the disease or because the suspected cases are not reported to National Institute of Health, Islamabad due to the fact that Karachi, the capital of the Sindh province, also has the diagnostic facilities

The people residing in these areas are also prone to vector borne diseases because they do not have access to vector control vaccines and other preventative strategies. Furthermore, animal husbandry is the major source of income for 70% of the population in KPK and FATA and the majority of people are living in poverty in these areas [35]. These rural-dwellers are unaware of animal pathogens and tick borne viruses and commonly live alongside their cattle with no preventive measures relating to hygiene, whatsoever. The families not only consume the milk and meat of their cattle to survive, but they also use the animals dung for wound healing with potentially dire consequences. The healthcare infrastructure in Pakistan is already under strain and poorly developed in these regions, so such practices compound significantly on the populations susceptibility to diseases like CCHF.

### Livestock and Eid-ul-Azha

Pakistan has a vast livestock population, owned by either commercial dairy farms and husbandries or the rural dwelling community. Currently farm animals in Pakistan comprise of 22.42 million cattle, 23.34 million buffalos, 49.14 million goats and 24.24 million sheep [37]. As a result, a large number of humans are in direct contact with these animals on a daily basis. These people perform a variety of tasks which can be a source of animal-to-human transmission of CCHFV, including; veterinary care, birthing procedures, removing ticks bare handed and animal slaughtering and butchery. People with such high exposure to cattle clearly have a greater risk of acquiring CCHV, as reported in a study from Afghanistan [36].

Unfortunately, people living in the high risk areas (for CCHF) of Pakistan are also involved in work practices that increase their risk. Furthermore, they routinely take their cattle to other regions of the country to sell them, mainly for consumption of meat, particularly at the time of Eid-ul-Azha (a festival in which Muslims sacrifice cattle to give away to others). As a result, a greater number of CCHF cases are reported around Eid-ul-Azha [38, 39]. The festival is celebrated according to the Islamic Lunar Calendar. In the past few years, the dates of Eid-ul-Azha have coincided with the winter season but in the coming decade, Eid-ul-Azha dates will fall in the summer season in Pakistan, thereby increasing the population growth of the ticks which carry the CCHFV [40]. The migration of infected animals to urban areas leads to transmission of CCHFV to other animals as well as to humans in those areas. The number of cases is expected to rise in the coming years due to these practices, unless strict preventive measures are taken and widespread awareness campaigns are launched.

Muslims purchase cattle and keep them at their houses for a few days before the Eid-ul-Azha festival. This poses a risk of transmission of CCHFV in humans via the bite of an infected tick. People usually perform the sacrifice of the animal themselves or appoint a butcher on Eid-ul-Azha day for slaughtering the animals at their homes. In both cases, no gloves or long sleeved shirts are used, as there is no formal training or awareness provided to either the people or the butchers. Thus, the likelihood that the person performing the sacrifice might acquire the infection from an animal is very high [35]. Furthermore, the butchers go from home to home to provide this service, and thus they can, in themselves, also be a source of transmission of the ticks and subsequent CCHFV to other humans.

Another problem is that after slaughtering the animals, the remains are thrown in open places and most cities do not have a proper system for disposal of offal. Apart from this domestic butchering, animals which die in road accidents or from natural illness are also sometimes left on the roads for weeks to rot. This flaw in the sanitary system regarding proper disposal of dead animals can lead to problems in addition to CCHF transmission. The Ministry of National Health Services, Pakistan issued a revised awareness letter in June 2015 focusing on the prevention of CCHFV spread. Unfortunately however it does not describe guidelines for animal slaughtering or control on migration of animals from high risk areas [40].

### Nomadic lifestyle

There are a number of tribes in Pakistan which lead a nomadic lifestyle, moving from one place to the other, looking for food and water. Pakistan is bestowed with a colossal river named the Indus River, which extends across the entire length of the country and drains into the Arabic ocean. Some tribes in Pakistan reside in tents along the banks of this river, and move as the weather and water supply shifts. The people of Cholistan, a desert in the Punjab province of Pakistan, also lead a semi-nomadic life, shifting from one place to the other to fulfil their needs of food gathering and water supply. Similarly, there are nomadic tribes present in the KPK, FATA and Baluchistan regions of Pakistan. Most of these tribes possess animals such as; goats, sheep, cows, buffalos and they also migrate along with them. These groups and their animals can be a real threat to the public health of Pakistan because they have the potential to transmit vector borne disease such as CCHF from endemic areas to what were previously safe areas. A study from Turkey reported that sleeping in the open air with animals during migrations is a major risk factor for CCHF infection [27]. It is also reported in another study that the CCHF outbreaks occur in the time periods following the movements of nomadic people and their cattle from their villages to district centres [35].

## Conclusion

Pakistani’s are at high risk of contracting CCHF and the reasons for this are outlined in this scoping review and include; ticks and the environment, the health care system, the rural community, livestock and Eid-ul-Azha and having a nomadic lifestyle. Some of these factors are cultural and others are physical, and they need to be addressed in different ways. The recent death of a prominent surgeon from an urban Pakistani hospital highlights the ongoing levels of transmission from rural to urban settings. It also highlights the need for robust strategies to be developed in-order to educate the general public, as well as Pakistani healthcare professionals about CCHFV. This would make people associated with the handling of livestock aware of the risks associated with animal handling and CCHFV transmission but would also help to halt the transmission of nosocomial infections. Checks and balances must be ensured with regards to the hygienic conditions of the Pakistani healthcare system.

The media can play a vital role in providing awareness about the modes of transmission and symptoms of CCHFV, usage of proper clothes to reduce contact with ticks in the process of cleaning animal folds and the importance of spraying animal folds in-order to protect them from attacks by ticks. Moreover, specialized workshops, informative seminars and educational programs are required for healthcare professionals. The government must refocus their energies and provide sufficient finances in-order to vaccinate agricultural livestock [29]. This is particularly important around the time of the festive occasion of Eid-ul-Azha. Establishing quarantine areas and provision of appropriate diagnostic facilities can also prevent the ongoing spread and consequently reduce the number of casualties from CCHFV. There is a dire need to control the migratory activities of people and animals from areas endemic with CCHF and to establish a registry and tracking system to monitor the movement of livestock across country borders and within the different provinces of Pakistan. Conducting epidemiological surveys to understand the tick fauna should also be mandatory. In this manner the “hotspots” of CCHFV and the risk factors associated with a particular region can be better identified. Such information can serve as useful evidence for various CCHFV control programs which can act as a supporting tool for public health campaigns that need to be implemented within endemic areas.

## Abbreviations

BHU: Basic Healthcare Units
BVH: Bahawalpur Victoria Hospital
CCHF: Crimean Congo Haemorrhagic Fever
CCHFV: Crimean Congo Haemorrhagic Fever Virus
FATA: Federally Administered Tribal Areas
KPK: Khyber Pakhtunkhwa
NIH: National Institute of Health
NWFP: North-West Frontier Province
